# Supplementary material for: Structural basis for substrate recognition mechanism of human SLC26A7
Source: Nat Commun. 2025 Aug 15;16:7600. doi: 10.1038/s41467-025-62792-w (PMC12356840; doi:10.1038/s41467-025-62792-w)
Supplement: Supplementary file 1 — Supplementary information [file 41467_2025_62792_MOESM1_ESM.pdf]

Supplementary Information

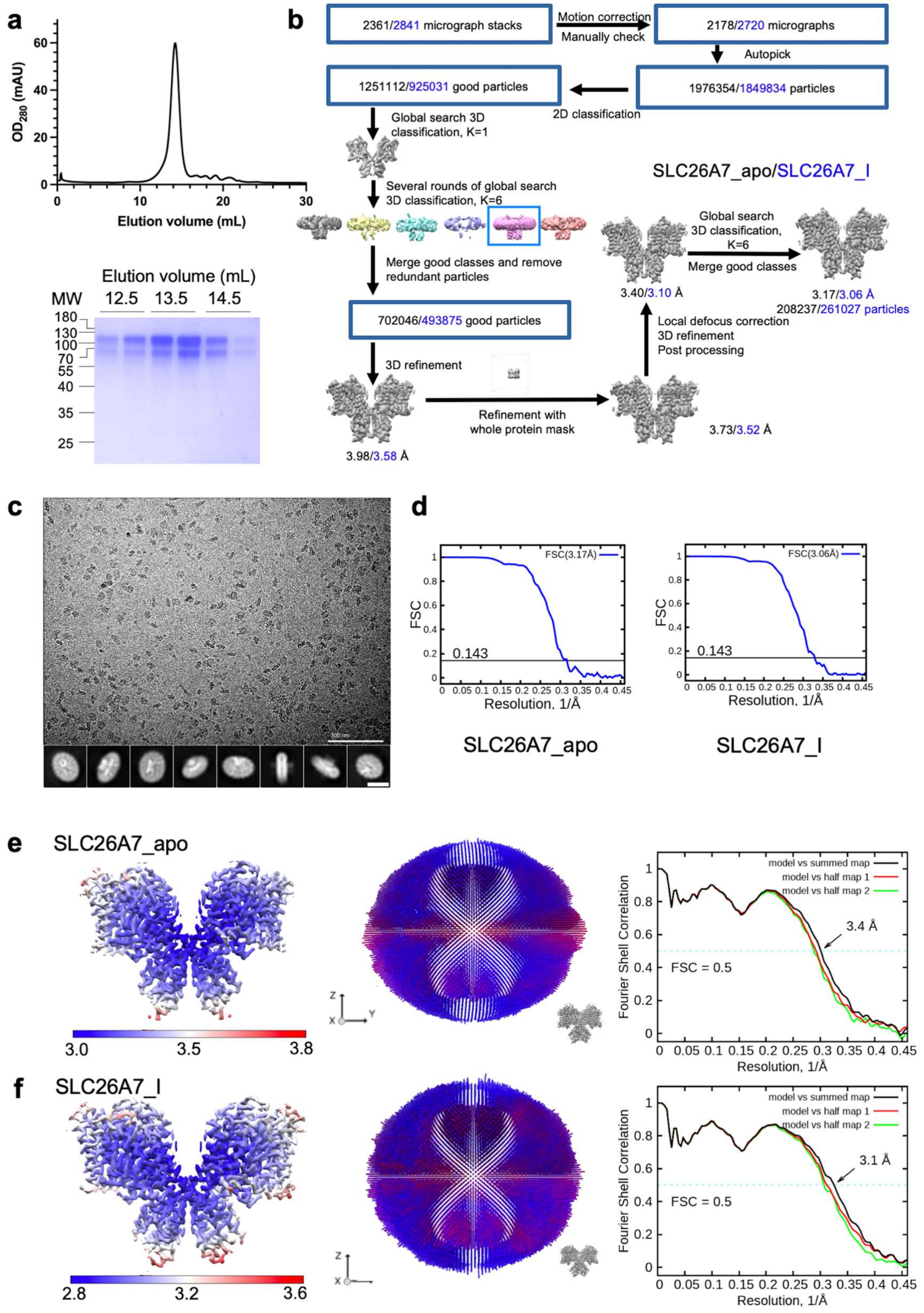

Supplementary Fig.1| Protein purification and cryo-EM data processing of human SLC26A7. a, Size exclusion chromatography results of human SLC26A7. Protein

migrates in larger molecular weight due to glycosylation. **b**, The flowchart for EM data processing of apo and iodide loading state SLC26A7 (labeled as SLC26A7_apo and SLC26A7_I, respectively). **c**, Representative electron micrograph and two-dimensional class averages results. **d**, Gold-standard Fourier shell correlation (FSC) curves for the 3D reconstruction of SLC26A7_apo and SLC26A7_I. **e**, Local resolution map (left) and Euler angle distribution (middle) for the 3D reconstruction of SLC26A7_apo. On the right is FSC curves of the refined model versus the overall map that was refined against (black), against the first half map versus the same map (red); and against the first half map versus the second half map (green). The small difference between the red and green curves indicates that the refinement of the atomic coordinates did not suffer from overfitting. **f**, Same as **e** but for SLC26A7_I.

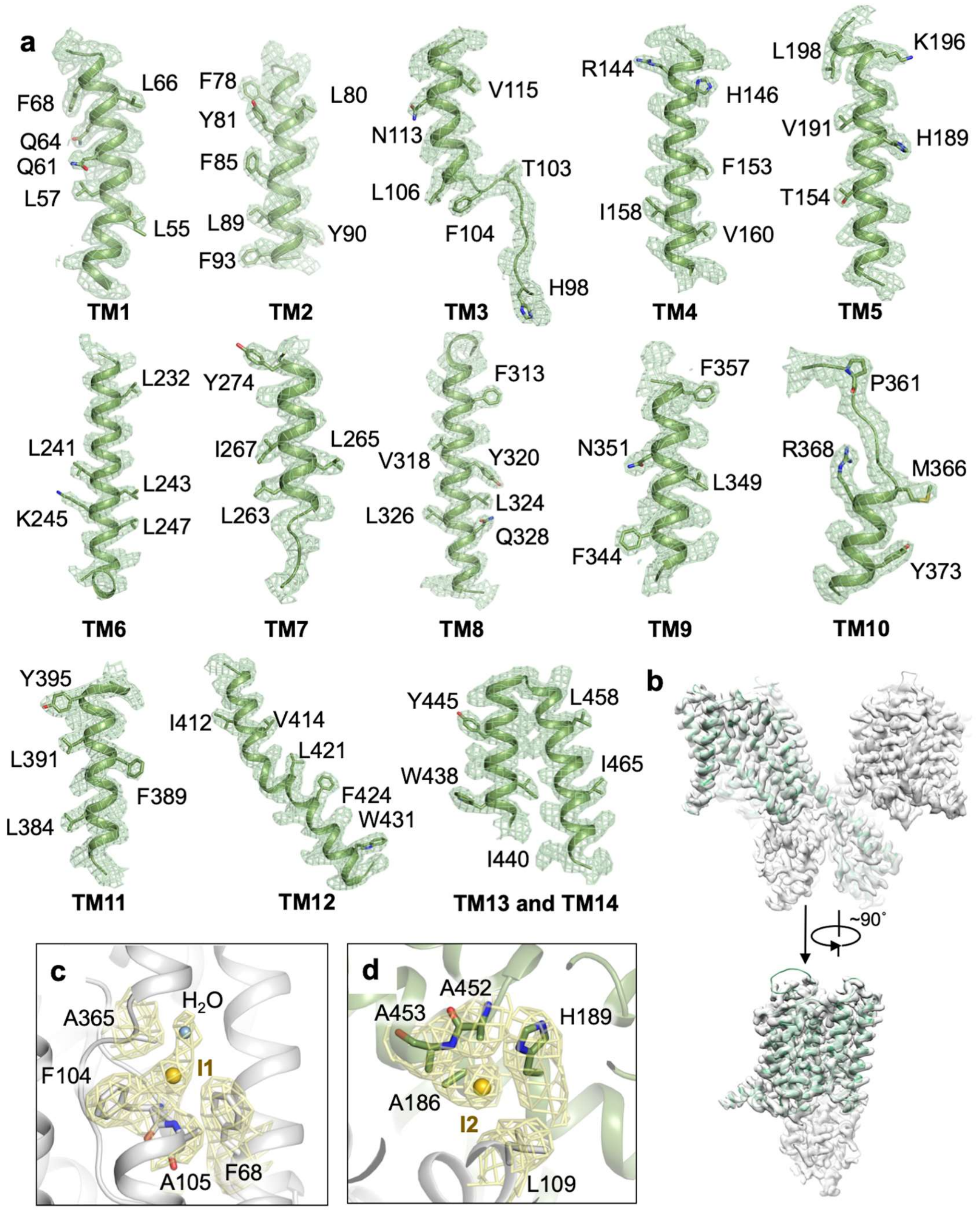

Supplementary Fig.2| Cryo-EM map quality of human SLC26A7 in iodide binding state. a, Cryo-EM map around the regions of SLC26A7 transmembrane helixes are shown in mesh. The side-chain of some amino acids are presented in sticks. The contour is 6σ. **b**, The model of SLC26A7 is fitted to cryo-EM map. Two different views are shown. **c**, The cryo-EM density around I1 binding pocket. **d**, The cryo-EM density around I2 binding pocket. The contour is 4σ in **c** and **d**.

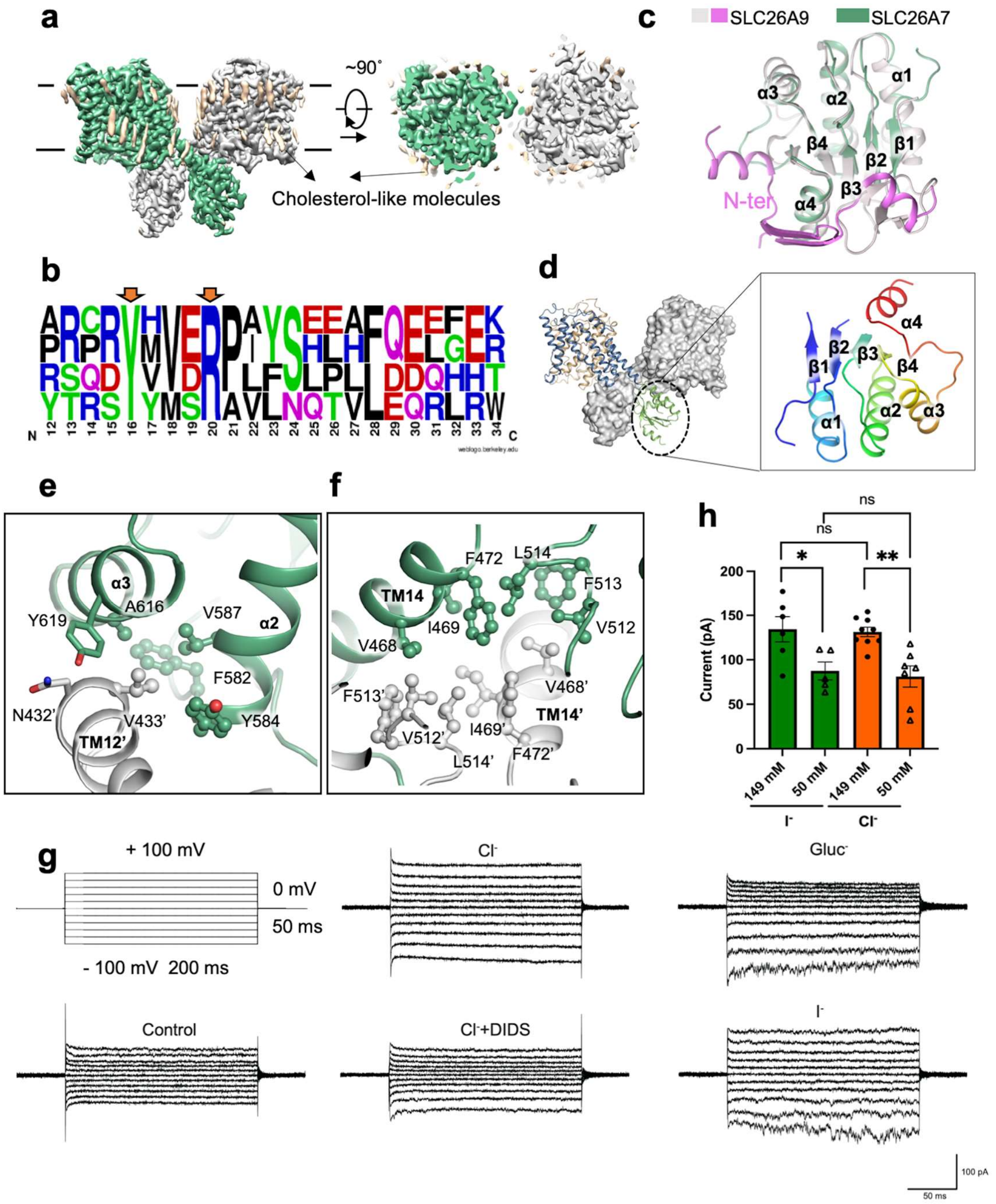

Supplementary Fig.3 | Domain arrangement of human SLC26A7. a, A series of cholesterol-like molecules are present around SLC26A7 TMD of cryo-EM map. **b**, Sequence conservation analysis of N-terminal beta sheet presenting in Pendrin, Prestin, SLC26A9, and SLC26A6[1]. The conserved tyrosine and arginine are important for structure integrality. **c**, Structure alignment of STAS domain of SLC26A9 and SLC26A7. N-terminal binding induces no deformation of STAS domain. **d**, STAS domain is placed under TMD from the other protomer. Enlarged panel shows the

detailed secondary structure of STAS in SLC26A7. **e**, Interface of STAS domain and gate domain. Hydrophilic interaction between Y619 and N432' is shown. The interaction between STAS domain and gate domain is further enhanced by internal hydrophobic network. **f**, Dimerization interface at the end of TM14 and α1-β1 loop of STAS domain is mainly constituted by hydrophobic interactions. **g**, Standard protocol and SLC26A7 representative recordings of step responses to Cl^-, Control , Cl^- with DIDS, $Gluc^-$ and I^- are shown. **h**, SLC26A7 transport activity in different ion concentrations. HEK293T cells transfected with SLC26A7 were used to measure the I^- and Cl^- current under different ion concentrations. Current under 149 mM ion concentration is obviously higher than that in 50 mM ion concentration both in the case of I^- and Cl^-. The number of cells patched for 149 mM I^-, 50 mM I^-, 149 mM Cl^-, 50 mM Cl^- were n =6, 5, 9, 7, respectively. Data represent the means ± SEM, *$P < 0.05$, **$P < 0.01$ (Significance is calculated by one-way ANOVA with Sidak test. The exact P values for each comparison are: 149 mM I^- vs. 149 mM Cl^-: 0.9992, 50 mM I^- vs. 50 mM Cl^-: 0.9902, 149 mM I^- vs. 50 mM I^-: 0.0292, 149 mM Cl^- vs. 50 mM Cl^-: 0.0039.).

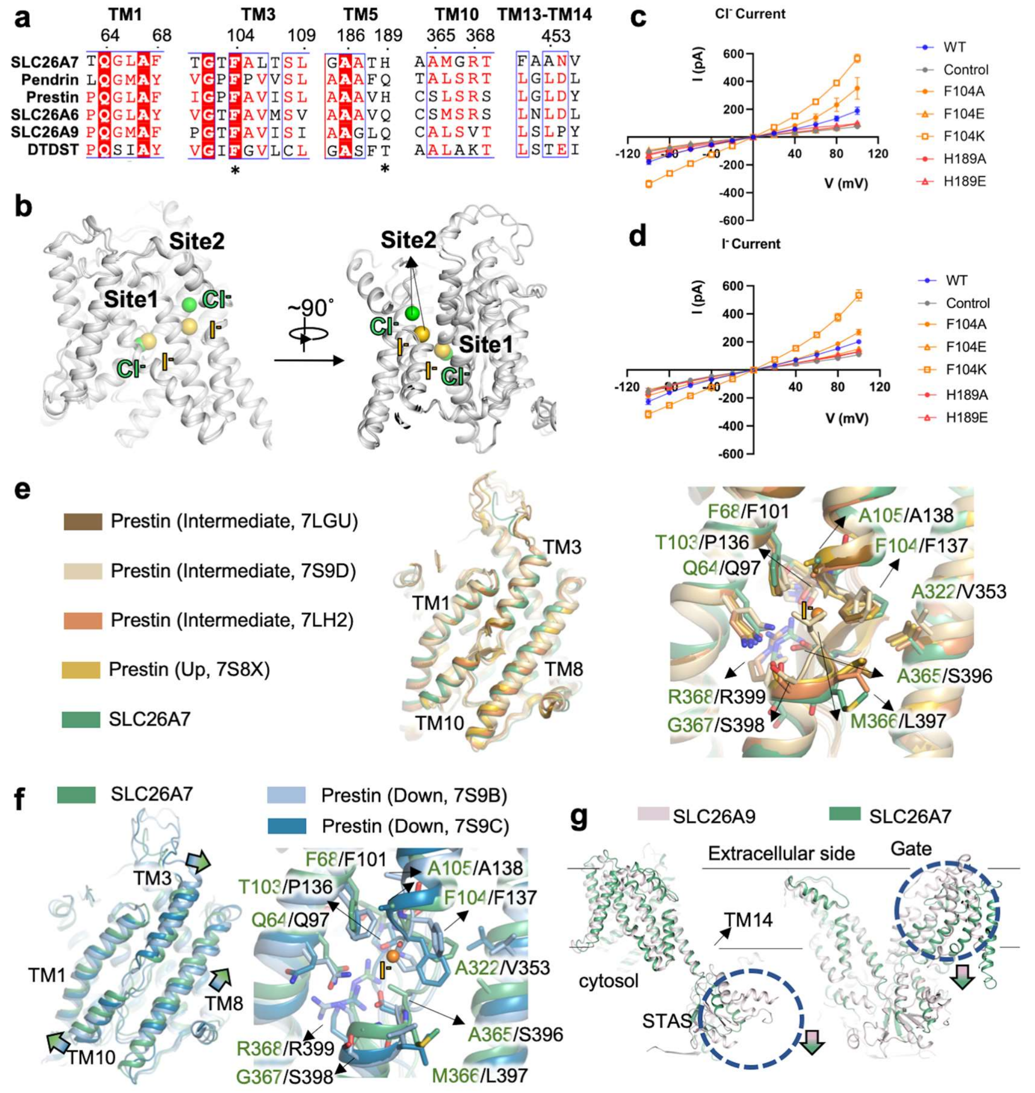

Supplementary Fig.4| Structure alignment and electrophysiological assay of key residues in I1 and I2 sites of SLC26A7. a, Sequence alignment of key residues in I1 and I2 sites. **b**, AlphaFold 3 predicted chloride binding in SLC26A7 is aligned with SLC26A7 iodide loading structure. Similar ion binding in I2 site is detected. **c**, HEK293T cells transfected with WT, control and mutants were used to measure the I/V curves of Cl^-. The number of cells patched for each group (in the order of WT, Control, F104A, F104E, F104K, H189A, H189E) were n = 17, 14, 14, 9, 19, 16, 11. **d**, Same as **c**, but for I^- current. The number of cells patched for each group are n = 29, 26, 20, 12, 16, 13, 27. Data represent the means ± SEM. **e**, Core domain alignment of SLC26A7_I and Prestin intermediate- and up-state structures. Zoom-in view around I1 binding site clearly exhibits conformational change of the ion coordination residues. **f**,

Core domain alignment of SLC26A7_I and Prestin down-state structures is shown. Deformation of TM3, TM8, and TM10 are displayed in the left. Residues conformational changes are enlarged in the right. Residues of SLC26A7 are labeled in green, while ones of Prestin are in black. **g**, Structural alignment of SLC26A9 and SLC26A7 with respect to TM14. STAS domain and gate domain move to the same direction supporting the close structural linkage.

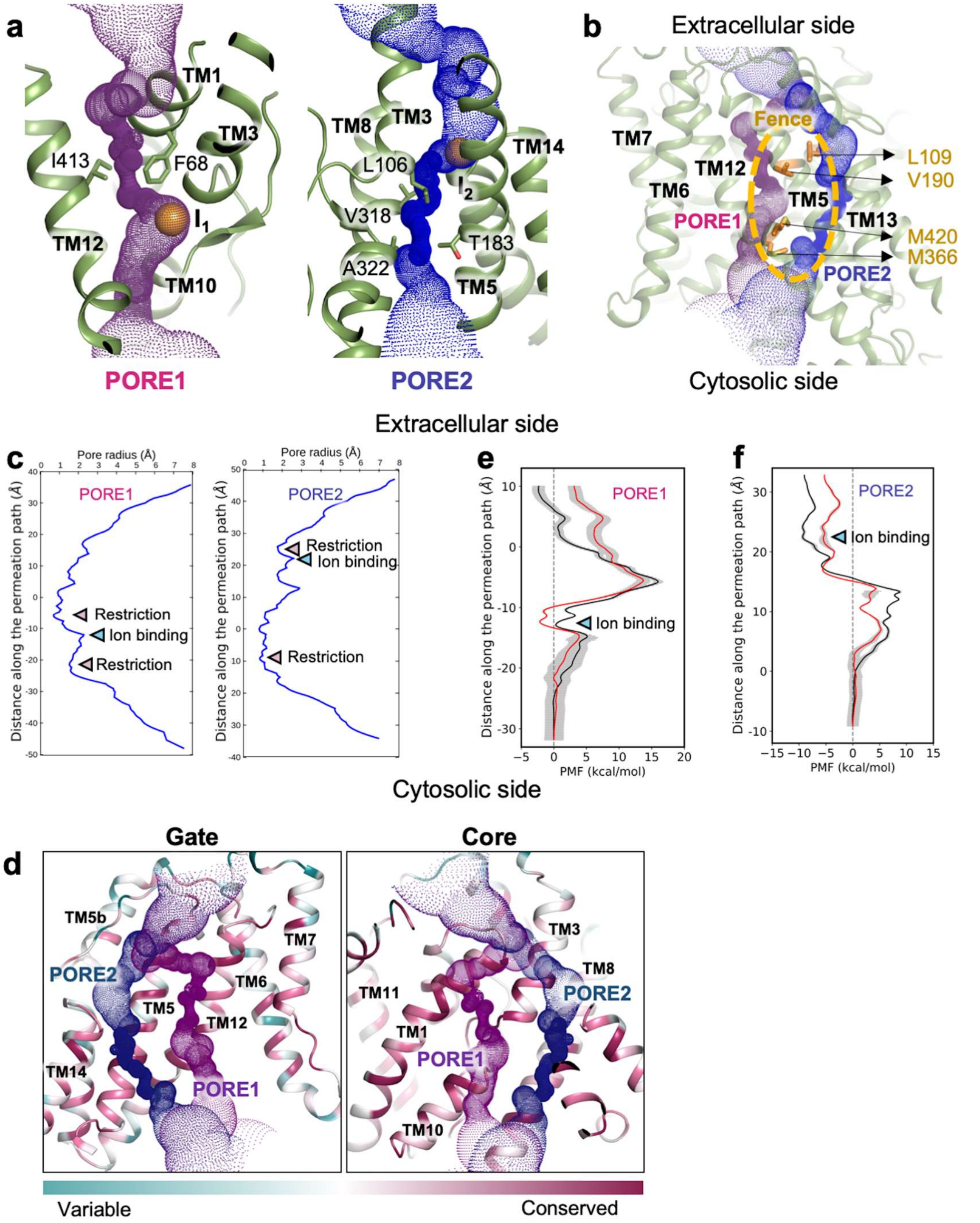

Supplementary Fig.5 | Two possible ion permeation paths are identified in human SLC26A7. a, PORE1 passing through I1 site is colored in purple. PORE2 passing through I2 site is colored in blue. **b**, The two ion-permeation paths are separated by hydrophilic fences. **c**, Radius measurement along PORE1 and PORE2 is shown. Ion binding and restriction sites are indicated by blue and pink triangles. **d**, Sequence conservation of residues around PORE1 and PORE2 is presented. Most residues located along the permeation path show high conservation. **e**, Umbrella sampling

results of PORE1 are shown. I1 site in PORE1 is global minimum site for both Cl^- and I^-. **f**, Umbrella sampling results of PORE2. I2 in PORE2 is global minimum only for I^-. The error bars were calculated using the block averaging method, where the trajectory data was divided into time blocks of 10 ns. The extracellular side and cytosolic side are the same in **c** and **e.** Ion biding sites are labeled. PMF curve for Cl^- is red, while that for I^- is black.

Supplementary Table 1 | Data and refinement statistics for SLC26A7 structures

	**SLC26A7_I**	**SLC26A7_Apo**
**PDB ID**	9IKV	9IKX
**EMDB**	EMD-60660	EMD-60662
**Data collection and Processing**		
Microscope	FEI Titan Krios	FEI Titan Krios
Voltage (kV)	300	300
Camera	K3	K3
Magnification	81000	81000
Pixel size at detector (Å/pixel)	1.0773	1.087
Total electron exposure (e-/Å^2)	50	50
Exposure rate (e-/pixel/sec)	23	23
Number of frames collected during exposure	32	32
Defocus range (μm)	1.0–2.0	1.0–2.0
Automation software	AutoEMation	AutoEMation
Energy filter slit width (eV)	20	20
Micrographs collected (no.)	2,841	2,361
Micrographs used (no.)	2,720	2,178
Total extracted particles (no.)	1,849,834	1,976,354
**Reconstruction**		
Refined particles (no.)	925,031	1,251,112
Final particles (no.)	493,875	208,237
Point-group	C2	C2
Resolution (global, Å)	3	3.2

FSC 0.5 (unmasked/masked)	3.75/3.47	3.84/3.61
FSC 0.143 (unmasked/masked)	3.28/3.06	3.45/3.17
Resolution range (local, Å)	2.8-3.6	3.0-3.8
Map sharpening B-factor (Å^2)	200	200
Map sharpening method	Relion	Relion
**Model composition**		
Protein (residues)	1132	1108
**Model refinement**		
Refinement package	Phenix 1.19	Phenix 1.19
- real or reciprocal space	Real space	Real space
-resolution cutoff	0.5	0.5
Model-Map scores		
-CC_volume/mask	0.85/0.89	0.86/0.87
B factors (Å^2)		
Protein residues	52.91	91.49
R.m.s deviations from ideal values		
Bonds length (Å)	0.003	0.002
Bonds Angle (°)	0.548	0.475
**Validation**		
MolProbity score	1.79	1.66
CaBLAM outliers (%)	4.4	2.6
Clashscore	5.58	4.62
Poor rotamers (%)	1.05	0.11
C-beta deviations (%)	0.00	0.00
EMRinger score	2.54	2.56
Ramachandran plot		
Preferred (%)	92.45	93.50
Allowed (%)	7.55	6.32
Outlier (%)	0.00	0.18

Summary table for MD simulations

**Reliability and reproducibility checklist for molecular dynamics simulations**<br>***All boxes must be marked YES by acceptance unless "Response not needed if No".**	**Yes**	**No**	**Response (Please state where this information can be found in the text)**
**1. Convergence of simulations and analysis**			
1a. Is an evaluation presented in the text to show that the property being measured has equilibrated in the simulations<br>(*e.g.* time-course analysis)?	☒	☐	Supporting information, page 3, paragraph 2.
1b. Then, is it described in the text how simulations are split into equilibration and production runs and how much data were analyzed from production runs?	☒	☐	Supporting information, page 3, paragraph 2.
1c. Are there at least 3 simulations per simulation condition with statistical analysis?	☒	☐	Supporting information, page 3, paragraph 2. Main text, page 13.
1d. Is evidence provided in the text that the simulation results presented are independent of initial configuration?	☒	☐	Main text, page 13.
**2. Connection to experiments**			
2a. Are calculations provided that can connect to experiments (*e.g.* loss or gain in function from mutagenesis, binding assays, NMR chemical shifts, J-couplings, SAXS curves, interaction distances or FRET distances, structure factors, diffusion coefficients, bulk modulus and other mechanical properties, *etc.*)?	☒	☐	Supporting information, page 3, paragraph 2.
**3. Method choice**			
3a. Do simulations contain membranes, membrane proteins, intrinsically disordered proteins, glycans, nucleic acids, polymers, or cryptic ligand binding?	☒	☐	Supporting information, page 3, paragraph 1.

3b. Is it described in the text whether the accuracy of the chosen model(s) is sufficient to address the question(s) under investigation (e.g. all-atom vs. coarse-grained models, fixed charge vs. polarizable force fields, implicit vs. explicit solvent or membrane, force field and water model, etc.)?	☒	☐	Supporting information, page 3, paragraph 2.
3c. Is the timescale of the event(s) under investigation beyond the brute-force MD simulation timescale in this study that enhanced sampling methods are needed?	☒	☐	Supporting information, page 3, paragraph 3.
If **YES**, are the parameters and convergence criteria for the enhanced sampling method clearly stated?	☒	☐	Supporting information, page 3, paragraph 3.
If **NO,** is the evidence provided in the text?	☐	☐	
**4. Code and reproducibility**			
4a. Is a table provided describing the system setup that includes simulation box dimensions, total number of atoms, total number of water molecules, salt concentration, lipid composition (number of molecules and type)?	☒	☐	Supporting information, page 3, paragraph 1.
4b. Is it described in the text what simulation and analysis software and which versions are used?	☒	☐	Supporting information, page 3, paragraph 1.
4c. Are other parameters for the system setup described in the text, such as protonation state, type of structural restraints if applied, nonbonded cutoff, thermostat and barostat, etc.?	☒	☐	Supporting information, page 3, paragraph 2.
4d. Are initial coordinate and simulation input files and a coordinate file of the final output provided as supplementary files or in a public repository?	☒	☐	Supporting information, page 5, Data availability.

4e. Is there custom code or custom force field parameters?		☒	☐	Supporting information, page 5, Data availability.
	If **YES**, are they provided as supplementary files or in a public repository?	☒	☐	Supporting information, page 5, Data availability.

References

1 Crooks, G. E., Hon, G., Chandonia, J. M. & Brenner, S. E. WebLogo: a sequence logo generator. *Genome Res* **14**, 1188-1190, doi:10.1101/gr.849004 (2004).
