# Supplementary material for: Structural basis for substrate recognition mechanism of human SLC26A7
Source: Nat Commun. 2025 Aug 15;16:7600. doi: 10.1038/s41467-025-62792-w (PMC12356840; doi:10.1038/s41467-025-62792-w)
Supplement: Supplementary file 2 — Description of Additional Supplementary Files [file 41467_2025_62792_MOESM2_ESM.pdf]

Description of Additional Supplementary Files

File Name: Supplementary Movie 1

Description: Conformational changes of different groups. Core domain movement during conformational change tandemly from inward, intermediate, to outward are shown by alignment to gate domain. Represented structures selected are as follows: Inward, SLC26A4 (PDB:7WLA, http://doi.org/10.2210/pdb7WLA/pdb) and SLC26A9 (PDB: 7CH1, http://doi.org/10.2210/pdb7CH1/pdb); Intermediate: SLC26A5 (PDB:7LGW) and SLC26A7 with substrate; Outward, SLC26A4 (PDB: 7WLB and chain B, http://doi.org/10.2210/pdb7WLB/pdb). For all structures, the chain A are selected otherwise indicated.
