# Supplementary material for: Structural basis for substrate recognition mechanism of human SLC26A7
Source: Nat Commun. 2025 Aug 15;16:7600. doi: 10.1038/s41467-025-62792-w (PMC12356840; doi:10.1038/s41467-025-62792-w)
Supplement: Supplementary file 4 — Reporting Summary [file 41467_2025_62792_MOESM4_ESM.pdf]

nature portfolio

Corresponding author(s): Ximin Chi

Last updated by author(s): Jun 18, 2025

Reporting Summary

Nature Portfolio wishes to improve the reproducibility of the work that we publish. This form provides structure for consistency and transparency in reporting. For further information on Nature Portfolio policies, see our Editorial Policies and the Editorial Policy Checklist.

Statistics

For all statistical analyses, confirm that the following items are present in the figure legend, table legend, main text, or Methods section.

n/a	Confirmed	
☐	☒	The exact sample size (*n*) for each experimental group/condition, given as a discrete number and unit of measurement
☐	☒	A statement on whether measurements were taken from distinct samples or whether the same sample was measured repeatedly
☐	☒	The statistical test(s) used AND whether they are one- or two-sided<br>*Only common tests should be described solely by name; describe more complex techniques in the Methods section.*
☐	☒	A description of all covariates tested
☐	☒	A description of any assumptions or corrections, such as tests of normality and adjustment for multiple comparisons
☐	☒	A full description of the statistical parameters including central tendency (e.g. means) or other basic estimates (e.g. regression coefficient) AND variation (e.g. standard deviation) or associated estimates of uncertainty (e.g. confidence intervals)
☐	☒	For null hypothesis testing, the test statistic (e.g. *F*, *t*, *r*) with confidence intervals, effect sizes, degrees of freedom and *P* value noted<br>*Give P values as exact values whenever suitable.*
☐	☒	For Bayesian analysis, information on the choice of priors and Markov chain Monte Carlo settings
☐	☒	For hierarchical and complex designs, identification of the appropriate level for tests and full reporting of outcomes
☐	☒	Estimates of effect sizes (e.g. Cohen's *d*, Pearson's *r*), indicating how they were calculated

Our web collection on statistics for biologists contains articles on many of the points above.

Software and code

Policy information about availability of computer code

Data collection	MotionCor2, AutoEMation, pClamp 11.2.2 software
Data analysis	Relion3, PyMol, UCSF Chimera, UCSF ChimeraX, PHENIX, COOT, MDFF, PROPKA, CHARMM-GUI, OpenMM, CHARMM36m, GraphPad Prism 9.5

For manuscripts utilizing custom algorithms or software that are central to the research but not yet described in published literature, software must be made available to editors and reviewers. We strongly encourage code deposition in a community repository (e.g. GitHub). See the Nature Portfolio guidelines for submitting code & software for further information.

Data

Policy information about availability of data

All manuscripts must include a data availability statement. This statement should provide the following information, where applicable:

- Accession codes, unique identifiers, or web links for publicly available datasets
- A description of any restrictions on data availability
- For clinical datasets or third party data, please ensure that the statement adheres to our policy

Atomic coordinates and EM maps have been deposited in the PDB (http://www.rcsb.org) and EMDB (https://www.ebi.ac.uk/pdbe/emdb), respectively. The PDB code for SLC26A7 in apo and iodide binding state is 9IKX and 9IKV respectively. The corresponding EMDB code is EMD-60662 and EMD-60660 respectively.

Research involving human participants, their data, or biological material

Policy information about studies with human participants or human data. See also policy information about sex, gender (identity/presentation), and sexual orientation and race, ethnicity and racism.

Reporting on sex and gender	This information has not been collected.
Reporting on race, ethnicity, or other socially relevant groupings	This information has not been collected.
Population characteristics	This information has not been collected.
Recruitment	This information has not been collected.
Ethics oversight	This information has not been collected.

Note that full information on the approval of the study protocol must also be provided in the manuscript.

Field-specific reporting

Please select the one below that is the best fit for your research. If you are not sure, read the appropriate sections before making your selection.

[x] Life sciences [ ] Behavioural & social sciences [ ] Ecological, evolutionary & environmental sciences

For a reference copy of the document with all sections, see nature.com/documents/nr-reporting-summary-flat.pdf

Life sciences study design

All studies must disclose on these points even when the disclosure is negative.

Sample size	No sample size calculation was performed. Sample size ranging from 5-35 was selected for electrophysiological assay. The sample size was selected to complish good signal-noise ratio.
Data exclusions	Some cells behaving resistance 5% lager than membrane resistance in electrophysiological assay are excluded. They are not suitable for current recording. The exclusion criteria is established before experiments.
Replication	All tempts at replications are successful.
Randomization	Allocation is random.
Blinding	Blinding is not necessary as cells are grouped by different plasmid transfection.

Reporting for specific materials, systems and methods

We require information from authors about some types of materials, experimental systems and methods used in many studies. Here, indicate whether each material, system or method listed is relevant to your study. If you are not sure if a list item applies to your research, read the appropriate section before selecting a response.

Materials & experimental systems

n/a	Involved in the study	
[x]	[ ]	Antibodies
[ ]	[x]	Eukaryotic cell lines
[x]	[ ]	Palaeontology and archaeology
[x]	[ ]	Animals and other organisms
[x]	[ ]	Clinical data
[x]	[ ]	Dual use research of concern
[x]	[ ]	Plants

Methods

n/a	Involved in the study	
[x]	[ ]	ChIP-seq
[x]	[ ]	Flow cytometry
[x]	[ ]	MRI-based neuroimaging

Eukaryotic cell lines

Policy information about cell lines and Sex and Gender in Research

Cell line source(s)	HEK293F, HEK293T
Authentication	Cell lines are purchased from Invitrogen.
Mycoplasma contamination	Cell lines are not tested for mycoplasma contamination.
Commonly misidentified lines (See ICLAC register)	No misidentified lines used in this study.

Plants

Seed stocks	No plant is used in this study.
Novel plant genotypes	No plant is used in this study.
Authentication	No plant is used in this study.
